# Sensitivity of Yeast Strains with Long G-Tails to Levels of Telomere-Bound Telomerase

**DOI:** 10.1371/journal.pgen.0030105

**Published:** 2007-06-22

**Authors:** Leticia R Vega, Jane A Phillips, Brian R Thornton, Jennifer A Benanti, Mutiat T Onigbanjo, David P Toczyski, Virginia A Zakian

**Affiliations:** 1 Department of Molecular Biology, Princeton University, Princeton, New Jersey, United States of America; 2 Cancer Research Institute, Department of Biochemistry and Biophysics, University of California, San Francisco California, United States of America; Yale University, United States of America

## Abstract

The Saccharomyces cerevisiae Pif1p helicase is a negative regulator of telomere length that acts by removing telomerase from chromosome ends. The catalytic subunit of yeast telomerase, Est2p, is telomere associated throughout most of the cell cycle, with peaks of association in both G1 phase (when telomerase is not active) and late S/G2 phase (when telomerase is active). The G1 association of Est2p requires a specific interaction between Ku and telomerase RNA. In mutants lacking this interaction, telomeres were longer in the absence of Pif1p than in the presence of wild-type *PIF1,* indicating that endogenous Pif1p inhibits the active S/G2 form of telomerase. Pif1p abundance was cell cycle regulated, low in G1 and early S phase and peaking late in the cell cycle. Low Pif1p abundance in G1 phase was anaphase-promoting complex dependent. Thus, endogenous Pif1p is unlikely to act on G1 bound Est2p. Overexpression of Pif1p from a non-cell cycle-regulated promoter dramatically reduced viability in five strains with impaired end protection *(cdc13–1*, *yku80*Δ, *yku70*Δ, *yku80–1*, and *yku80–4),* all of which have longer single-strand G-tails than wild-type cells. This reduced viability was suppressed by deleting the *EXO1* gene, which encodes a nuclease that acts at compromised telomeres, suggesting that the removal of telomerase by Pif1p exposed telomeres to further C-strand degradation. Consistent with this interpretation, depletion of Pif1p, which increases the amount of telomere-bound telomerase, suppressed the temperature sensitivity of *yku70*Δ and *cdc13–1* cells. Furthermore, eliminating the pathway that recruits Est2p to telomeres in G1 phase in a *cdc13–1* strain also reduced viability. These data suggest that wild-type levels of telomere-bound telomerase are critical for the viability of strains whose telomeres are already susceptible to degradation.

## Author Summary

Telomeres, the ends of linear chromosomes, are essential for chromosome stability. Telomerase is the enzyme that is responsible for lengthening telomeres in most organisms, including humans. One mechanism of survival for many human cancers is increased expression of telomerase. In baker's yeast, telomerase acts only late in the cell cycle, even though the catalytic subunit of telomerase is telomere bound throughout most of the cell cycle. Pif1p is a yeast helicase that limits telomerase by removing it from DNA ends. We demonstrate that Pif1p abundance is cell cycle regulated with its highest expression at the same time when telomerase acts. Consistent with this expression pattern, Pif1p is able to remove the active form of telomerase from DNA ends. Reducing the amount of telomere-bound telomerase either by Pif1p overexpression or by mutation in strains with defective telomere end protection causes death. Moreover, reducing Pif1p levels in the same end protection mutants improves their growth. These experiments suggest that compared to wild-type cells, strains with defective end protection require more telomere-bound telomerase for the proper replication or proper protection of their chromosome ends.

## Introduction

Telomeres, the physical ends of linear chromosomes, are required for the complete replication and stable maintenance of eukaryotic chromosomes. They are comprised of stretches of repeated C/G-rich DNA with the G-rich strand extended to form a 3′ single-stranded tail (G-tail). In *S. cerevisiae,* chromosomes end in ~300 base pairs of C_1–3_A/TG_1–3_ DNA. Although yeast telomeres bear short G-tails throughout most of the cell cycle [1], long G-tails occur transiently in late S/G2 phase [2].

As in many organisms, yeast telomeric DNA is assembled into a non-nucleosomal chromatin structure called the telosome [3]. The essential protein Cdc13p is a telosome component that binds the single-stranded G-tail and is required to protect telomeric DNA from degradation (reviewed in [4,5]). Likewise, the heterodimeric Ku complex, encoded by *YKU70* and *YKU80,* is telomere associated [6] and helps protect telomeres from degradation and from inappropriate recombination [7–9]. When yeast cells are limited for Cdc13p or for the Ku complex, they have long single-stranded G-tails [6,10]. Disruption of the 5′ to 3′ exonuclease *EXO1* in *yku80*Δ and *cdc13–1* strains suppresses the formation of these long G-tails [8,11]. Thus, Cdc13p and Ku are both required for telomere integrity. In their absence, telomeres are more prone to C-strand degradation mediated by Exo1p and perhaps other nucleases.

From yeasts to humans, telomeric DNA is replicated by a specialized reverse transcriptase called telomerase. In *S. cerevisiae, EST2* encodes the catalytic subunit of telomerase, *TLC1* encodes the templating RNA, and *EST1* encodes a telomerase subunit that functions in recruitment and perhaps activation of telomerase (reviewed in [4]). Yeast telomerase action is cell cycle regulated: it is able to lengthen telomeres only in the late S/G2 phase even though telomerase activity can be detected by in vitro assays in extracts prepared from cells at other times in the cell cycle [12,13]. However, Est2p is telomere associated throughout most of the cell cycle [14], including G1 and early S phase when telomerase is not active [12,13]. The telomere binding of Est2p in G1 phase requires a specific interaction between *TLC1* RNA and Yku80p, an interaction that is eliminated in both *tlc1*Δ*48* and *yku80*-*135i* mutants [15–17]. Since telomeres are short but stable in *yku70*Δ, *yku80*Δ, *tlc1*Δ*48,* and *yku80-135i* cells, the G1 recruitment of Est2p contributes to, but is not essential for, telomere length maintenance. The Ku-TLC1 interaction also contributes to the formation of new telomeres at double-strand breaks [17].

There is a second peak of Est2p association in late S/G2 phase that occurs at the same time in the cell cycle when Cdc13p binding increases, Est1p binding occurs [14], and telomerase acts [12,13]. This late S/G2 peak of Est2p binding is lost in the telomerase defective *cdc13–2* strain [14] as well as in several other telomerase deficient strains, such as an *est1*Δ strain (K.M. Daumer, J.B. Boulé, A. Chan, T.S. Fisher, and V.A. Zakian, unpublished data). In contrast, in *tlc*Δ*48, yku80*-*135i,* and *yku*Δ cells, Est2p and Est1p are telomere associated in late S/G2 phase, albeit at reduced levels relative to wild-type cells [16]. Thus, there are two pathways that recruit Est2p to telomeres. Although both pathways contribute to telomere maintenance, the late S/G2 peak is necessary and sufficient for telomerase-mediated lengthening. The mechanism by which the G1 phase Est2p binding contributes to telomere maintenance is not known.

The Pif1p helicase, a member of a helicase family conserved from yeasts to humans (reviewed in [18]), was first identified because of its role in the maintenance of yeast mitochondrial DNA [19]. However, cells also express a nuclear form of Pif1p that is a negative regulator of telomerase [20–22]. The telomeric role of Pif1p was discovered because of its strong inhibition of telomerase-mediated telomere addition to double-strand breaks [20]. This inhibition contributes to genome stability as gross chromosomal rearrangements such as translocations and deletions are elevated ~1,000-fold in *pif1*Δ cells [23]. Pif1p also affects the lengths of existing telomeres: reduced Pif1p causes telomere lengthening, while overexpressed Pif1p results in modest telomere shortening [20,21]. The effects of Pif1p on telomere addition and lengthening of existing telomeres require both telomerase and the helicase activity of Pif1p, indicating that Pif1p acts catalytically to inhibit telomerase [21,23]. Indeed, in vitro Pif1p reduces the processivity of telomerase and releases active telomerase from telomeric oligonucleotides [22]. In vivo, overexpression of Pif1p reduces Est2p and Est1p association with telomeres, whereas depletion of Pif1p increases the levels of telomere-bound Est1p [22], the telomerase subunit that is present on the telomere only when telomerase is active [14]. Thus, Pif1p helicase activity limits telomerase action both in vivo and in vitro by displacing active telomerase from DNA ends.

In addition to reducing the levels of telomere-bound telomerase, overexpression of Pif1p results in increased telomeric association of Cdc13p [22]. Since increased Cdc13p binding occurs when telomeres have long G-tails, as in a *yku*Δ strain [16] or in late S/G2 phase [14], these data suggest that unscheduled Pif1p-mediated removal of telomerase impairs the structural integrity of the telomere. Here we show that the abundance of nuclear Pif1p is cell cycle regulated with peak abundance late in the cell cycle when telomerase is active. Overexpression of Pif1p reduced the viability of *yku*Δ and *cdc13–1* cells. This reduced viability was suppressed by mutations in *EXO1,* suggesting that the decreased viability was due to an exacerbation of the reduced end protection that occurs in these mutants upon removal of telomerase from telomeres that are already deficient in end protection. When the *tlc1*Δ*48* or *yku80-135i* mutations were used to eliminate the G1 phase pathway for telomerase recruitment in *cdc13–1* cells, the temperature-sensitive phenotype of this strain worsened. Limiting expression of nuclear Pif1p to late in the cell cycle may enhance the protective role of yeast telomerase by allowing enzymatically inactive telomerase to remain telomere associated throughout much of the cell cycle.

## Results

### Pif1p Expression Is Cell Cycle Regulated

**Figure 1:**
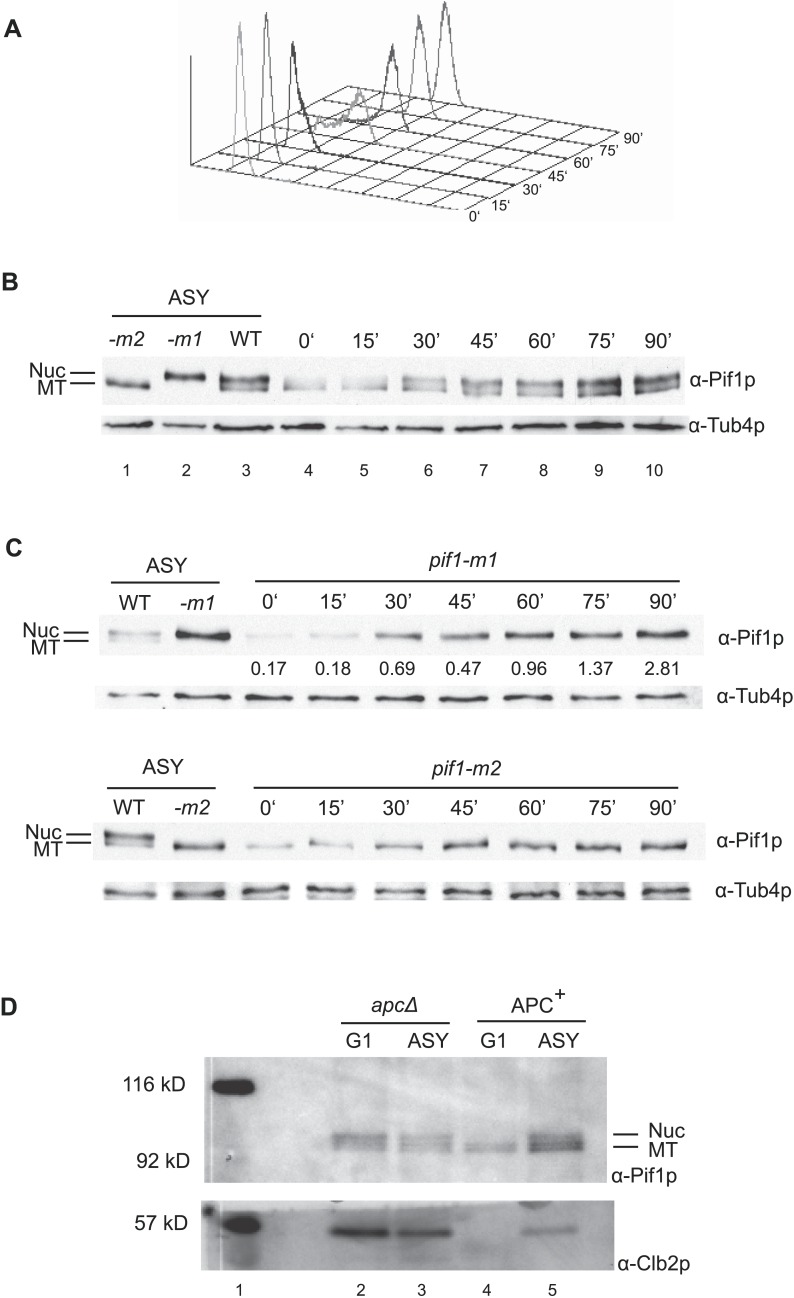
Pif1p Abundance Is Cell Cycle Regulated and APC Dependent (A and B) Wild-type cells were arrested in alpha factor at 24 °C and released into the cell cycle by the addition of pronase. At the indicated times, cells were harvested for FACS analysis to monitor DNA content (A) and by western blot analysis using anti-Pif1p antibodies to determine levels of nuclear (Nuc) and mitochondrial (MT) Pif1p and then with anti-gamma tubulin antibodies as a loading control (B). Pif1p abundance was also determined in asynchronous (ASY) *PIF1* (WT), *pif1-m2* (which express only mitochondrial Pif1p; -*m2*), and *pif1-m1* (which express only nuclear Pif1p; -*m1*) cells. (C) *pif1-m1* (top panel) or *pif1-m2* cells were synchronized as in (A), and levels of nuclear or mitochondrial Pif1p or gamma-tubulin monitored by western blotting. To estimate the relative abundance of nuclear versus mitochondrial Pif1p, the amount of signal in nuclear or mitochondrial Pif1p was determined by quantitating scanned blots using Scion Image, and then this value was normalized to the amount of gamma-tubulin in the same sample. The numbers under the *pif1-m1* western refer to the ratio of normalized nuclear Pif1p to normalized mitochondrial Pif1p at each time point. (D) Western blots of extracts from unbudded G1 phase cells (obtained by elutriation) and asynchronous (ASY) *apc*Δ or APC^+^ cells are shown after analysis with anti-Pif1p antibodies (top) or anti-Cbl2p antibodies (lower panel).

To determine if Pif1p levels vary during the cell cycle, wild-type cells were arrested in late G1 phase with alpha factor and released synchronously into the cell cycle. Cell cycle progression was monitored by fluorescence-activated cell sorting (FACS) (Figure 1A). Whole-cell protein extracts were prepared from samples taken throughout the cell cycle and analyzed by SDS-PAGE and western blotting using anti-Pif1p antibodies (Figure 1B). As previously demonstrated [21], there are two major forms of Pif1p in cells (Figure 1B, lane 3). The larger form, which is localized to nuclei, is the only form in cells carrying the *pif1-m1* allele [20,21] (Figure 1B, lane 2). The smaller form, which is generated by proteolysis during its import into mitochondria, is present in *pif1-m2* cells (Figure 1B, lane 1). A minor species of somewhat slower mobility, which may be the uncleaved precursor of the mature mitochondrial form, is often visible in *pif1-m2* and *PIF1* (but not *pif1-m1*) cells (see also [24]).

In wild-type cells, both the nuclear (Nuc) and mitochondrial (MT) forms of Pif1p were present in asynchronous cells (Figure 1B, ASY WT). However, the nuclear form was present in very low amounts in extracts from late G1 phase cells (Figure 1B, 0- and 15-min time points) but increased in abundance as cells progressed through the cell cycle (Figure 1B, time points 30–90 min). This cell cycle regulated pattern was confirmed by examining Pif1p abundance in *pif1-m1* cells, which express only the nuclear form of Pif1p [20,21]. Pif1p-m1 was barely detectable at 0 and 15 min but increased in abundance as cells progressed through S phase (Figure 1C, *pif1-m1*). Mitochondrial Pif1p was also less abundant in G1 and early S phase than later in the cell cycle, but not to the same extent as Pif1p-m1 (Figure 1C, *pif1-m2*). Quantitation of the gels in Figure 1C indicates that the relative abundance of nuclear to mitochondrial Pif1p was low (0.2) in G1/early S phase and high late in the cell cycle (~3).

### Steady State Levels of Nuclear Pif1p Are higher in Strains Lacking the Anaphase-Promoting Complex

Protein levels for mitochondrial Pif1p were higher than for nuclear Pif1p in late G1 phase cells (Figure 1B and 1C, 0- and 15-min time points). This difference suggested that the compartmentalization of Pif1p into the mitochondria may protect it from degradation. Since the anaphase-promoting complex (APC) is active from anaphase through late G1 phase [25–27], we considered that nuclear Pif1p might be targeted for destruction by the APC. To test this possibility, we examined Pif1p levels in APC^+^ and *apc*Δ strains (Figure 1D). Although the yeast APC is essential, its essential functions can be bypassed by deleting two of its targets *(PDS1* and *CLB5)* and overexpressing the Clb/CDK inhibitor, Sic1p [28]. We compared the levels of Pif1p in extracts from G1 cells obtained by elutriation and from cycling cells in both APC^+^ and in *apc*Δ strains by western blot analysis using anti-Pif1p antibodies. As a control, levels of Clb2p were also assayed (Figure 1D). As expected, Clb2p was detected in extracts from G1 phase *apc*Δ but not from G1 phase APC^+^ cells (Figure 1D, lanes 2 and 4). In the same extracts, the pattern of expression for nuclear Pif1p was similar to that of Clb2p; Pif1p was present in G1 phase cells in the *apc*Δ but not in the APC^+^ strain. In contrast, mitochondrial Pif1p was detectable in both G1 phase and asynchronous cells from both strains (Figure 1D, lanes 2–5). Furthermore, nuclear Pif1p was stabilized in alpha-factor arrested *cdc23–1* cells at semipermissive temperatures (L.R. Vega and V.A. Zakian, unpublished data). As Cdc23p is an essential subunit of the APC [29,30], these results also indicate that the abundance of nuclear Pif1p is APC dependent.

We also examined the stability of Pif1p in asynchronous APC^+^ and *apc*Δ cells after treatment with cycloheximide for various lengths of time (Figure S1). In the presence or absence of cycloheximide, the steady state levels of Pif1p were higher in *apc*Δ than in APC^+^ cells. Similar results were seen with the known APC target Clb2p but not for Cdc28p, a protein that is not turned over by the APC. Furthermore, during cycloheximide treatment, there was a modest, but reproducible increase in the half-life of Pif1p in *apc*Δ versus APC^+^ cells. Taken together, these experiments indicate that Pif1p levels are APC dependent.

### Pif1p Acts on Late S/G2 Phase Telomerase

The reduced telomere association of Est2p seen upon Pif1p overexpression [22] could be due to Pif1p affecting Est2p association with telomeres in G1 phase, late S/G2 phase, or both. The G1 telomere association of Est2p requires a specific interaction between the heterodimeric Ku complex and TLC1 RNA that is eliminated in *yku70*Δ, *tlc1*Δ*48,* or *yku80-135i* strains [16,17]. To determine if Pif1p affects the late S/G2 phase activity of telomerase, we examined telomere length in *pif1*Δ strains that also lacked G1 bound telomerase. If Pif1p acts on late S/G2 phase telomerase, we anticipate that telomeres will be longer in the double mutant strains than in the single mutant cells.

**Figure 2:**
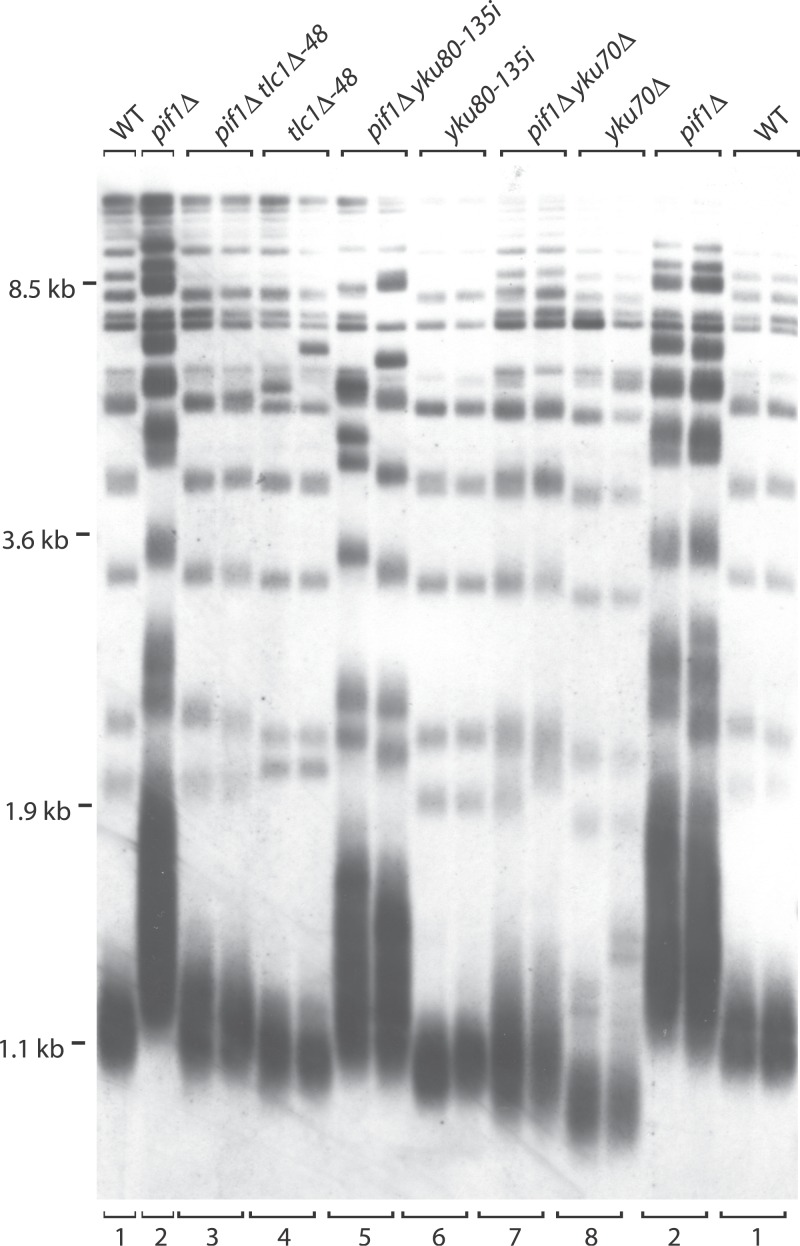
Pif1p Acts on Telomerase That Is Telomere Associated in Late S/G2 Phase DNA from the indicated strains was prepared from individual spore clones grown for ~30 generations after sporulation, digested with *Xho*I, separated on 1.0% agarose gels, and analyzed by Southern blotting using a C_1−3_A/TG_1−3_ telomeric probe. Telomere length was determined in WT (lanes 1) and *pif*1Δ (lanes 2) cells as well as in three mutant strains in which Est2p is not telomere bound in G1 phase (*tlc1*Δ*48,* lanes 4; *yku80-135i,* lanes 6; *yku70*Δ*,* lanes 8). In each of these strains, eliminating Pif1p results in longer telomeres than in the respective single mutant (*tlc1*Δ*48 pif1*Δ*,* lanes 3; *yku80-135i pif1*Δ*,* lanes 5; *yku70*Δ *pif1*Δ*,* lanes 7). For each genotype, telomere length is shown for two independent spore clones.

Diploids heterozygous for *pif1*Δ and either *tlc1*Δ*48, yku80-135i*, or *yku70*Δ were sporulated and dissected. Single and double mutants were identified and assayed for telomere length by Southern blot analysis (Figure 2). As expected, *pif1*Δ spore clones (Figure 2; lanes labeled 2) had longer telomeres than wild type (lanes 1), while the *tlc1*Δ*48* (lanes 4), *yku80-135i* (lanes 6), and *yku70*Δ (lanes 8) strains had short telomeres. In each case, the *pif1*Δ double mutants had longer telomeres than the corresponding single mutants. For example, telomeres were longer in *pif1*Δ *yku80-135i* cells (lanes 5) than in *yku80-135i* cells (lanes 6). Thus, endogenous Pif1p must be able to act on Est2p that is recruited to the telomere in late S/G2 phase since *tlc1*Δ*48*, *yku80-135i,* and *yku70*Δ cells lack G1 bound telomerase [16]. However, Pif1p may also be able to remove Est2p that is telomere bound at other times in the cell cycle since the *PIF1* deletion had a smaller effect on telomere length in the *tlc1*Δ*48* and *yku70*Δ backgrounds (but not in *yku80-135i* cells) than in wild-type cells.

There are no known separation-of-function alleles of *TLC1*, *YKU,* or other genes that eliminate telomerase binding in late S/G2 while retaining the ability to maintain telomeres by telomerase. Thus, it is not possible to do an experiment comparable to that in Figure 2 to determine if Pif1p removes Est2p from telomeres in G1 phase. However, since levels of nuclear Pif1p were low in G1 phase cells (Figure 1), endogenous Pif1p likely acts primarily on the active telomerase that is telomere associated in late S/G2 phase.

### Pif1p Overexpression Is Lethal in Strains Lacking Ku70p

Overexpression of Pif1p results in higher levels of telomere-bound Cdc13p, suggesting that Pif1p overexpressing cells have longer or more prolonged G-tails than wild-type cells [22]. These data led us to consider that end protection might be compromised in Pif1p overexpressing cells due to the unscheduled removal of telomerase from chromosome ends. If this model is correct, overexpression of Pif1p from the non-cell cycle-regulated *GAL1* promoter might compromise the growth of strains such as *yku*Δ that already have defects in end protection [7,8,31].

**Figure 3:**
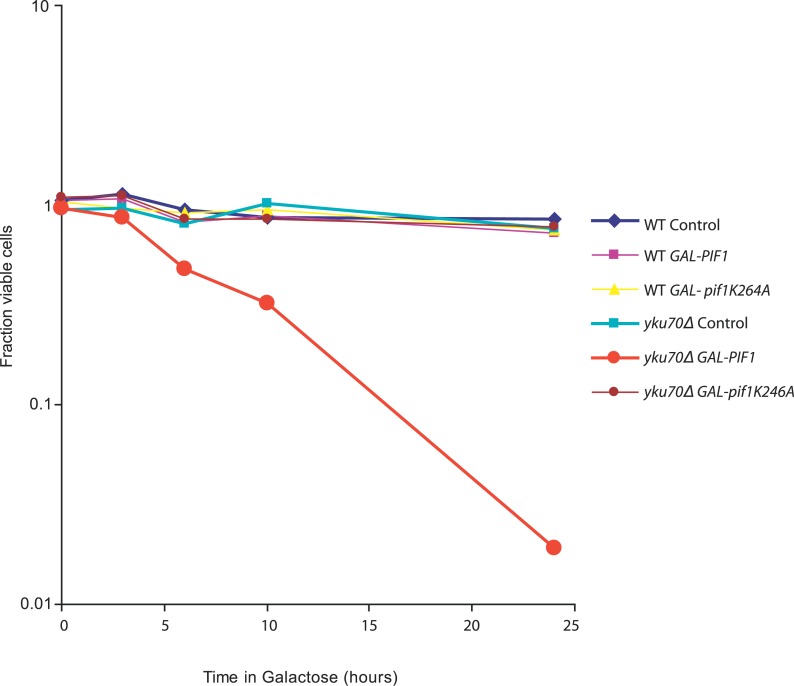
Pif1p Overexpression Is Lethal in *yku70*Δ Cells Wild-type (WT) or *yku70*Δ cells carried either vector alone (control) or the same vector with either WT Pif1p *(GAL-PIF1)* or catalytically inactive Pif1p *(GAL*-*pif1K264A),* both expressed from the galactose inducible *GAL1* promoter. Cells were grown overnight in 3% raffinose synthetic medium lacking leucine. At T = 0, galactose was added to a final concentration of 3% to induce protein expression. At the indicated time points, viability of the culture was monitored by plating cells onto glucose plates lacking leucine. Plates were counted after 2 d of growth at 30 °C.

**Figure 4:**
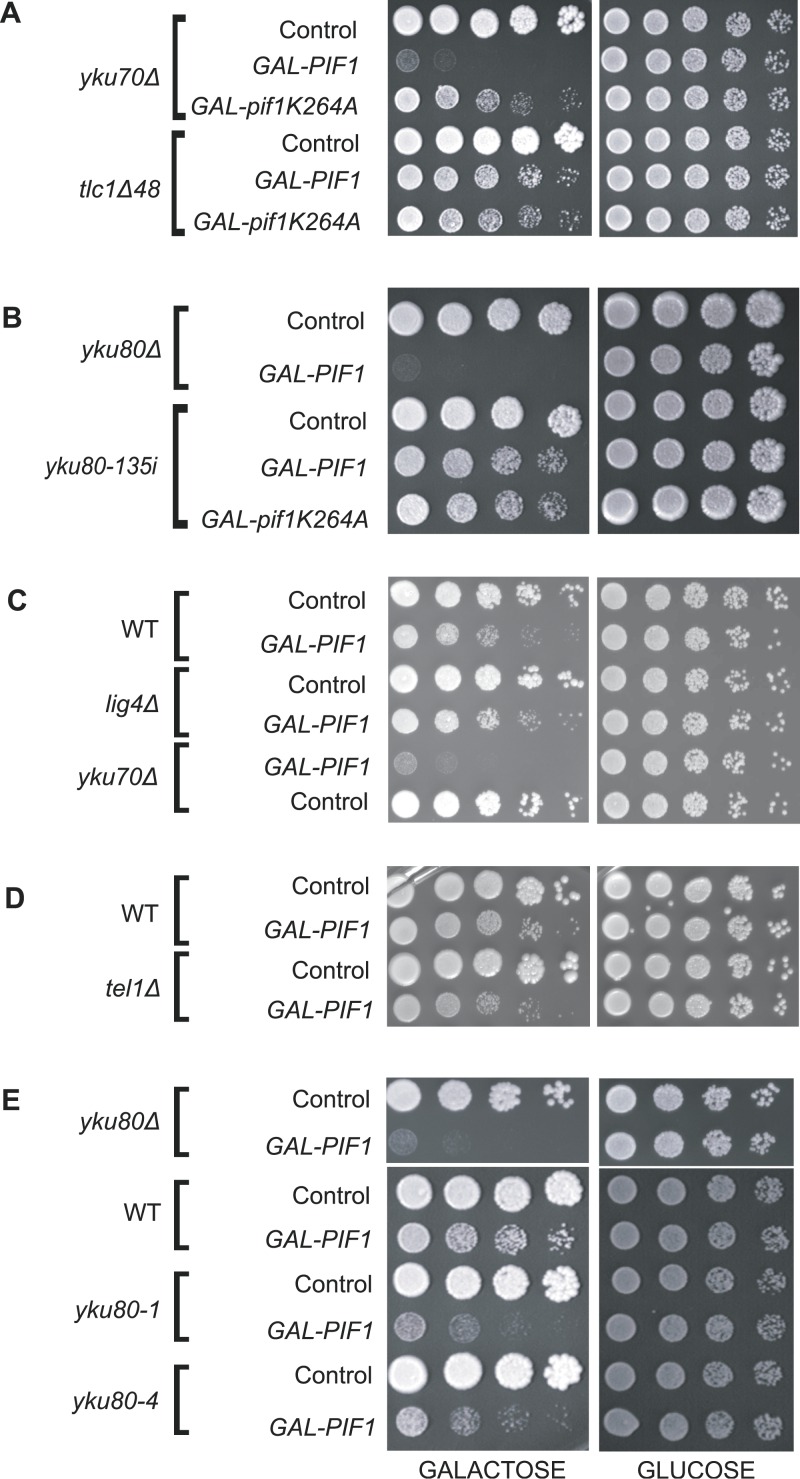
Pif1p Overexpression Is Lethal Only in Mutants Defective in Telomere End Protection Strains containing the indicated plasmids (described in Figure 3 legend) were grown overnight in 2% glucose synthetic liquid medium lacking either leucine (A–D) or leucine and tryptophan (E). Cultures were serially diluted in water and spotted onto plates containing either galactose (left) or glucose (right). Plates were photographed after 4 or 5 d (galactose) or 2 d (glucose) at 30 °C. (A) *yku70*Δ and *tlc1*Δ*48*; (B) *yku80*Δ and *yku80-135i*; (C) WT, *lig4*Δ, and *yku70*Δ; (D) WT and *tel1Δ;* (E) *yku80*Δ, WT, *yku80–1*, and *yku80–4*.

**Figure 5:**
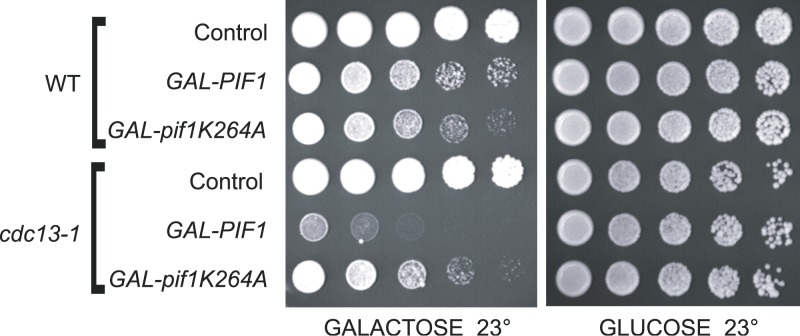
Overexpression of Pif1p Decreases Viability of *cdc13–1* Cells at Otherwise Permissive Temperatures Wild-type or *cdc13–1* strains containing the indicated plasmid (as described in Figure 3 legend) were grown overnight at 23 °C in synthetic liquid glucose medium lacking leucine. Cultures were serially diluted in water, spotted onto plates lacking leucine and containing either galactose (left) or glucose (right), and incubated at 23 °C for 7 d (galactose) or 5 d (glucose).

To test this possibility, wild-type and *yku70*Δ strains were transformed with an empty plasmid or the same plasmid containing wild-type *PIF1* or the catalytically inactive *pif1*-*K264A* allele. Both the wild-type and the inactive *pif1*-*K264A* alleles were expressed under the control of the galactose inducible *GAL1* promoter [22]. The viability of the strains after galactose induction at 30 °C was monitored by plating aliquots of the galactose grown culture back to glucose-containing plates (Figure 3). Overexpression of Pif1p in wild-type cells conferred a very modest loss of viability (Figure 3, squares). In contrast, overexpression of Pif1p in *yku70*Δ cells resulted in a dramatic loss of viability (Figure 3, large circles). This lethality required the helicase activity of Pif1p, since the viability of *yku70*Δ (Figure 3, small circles) and wild-type cells (Figure 3, triangles) was unaffected by overexpressing the helicase inactive Pif1p-*K264A*. However, overexpression of Pif1p-*K264A* did result in slow growth, as reflected in reduced colony size in both wild-type and *yku70*Δ cells (see Figures 4 and 5). Overexpression of wild-type Pif1p also caused lethality in *yku80*Δ cells (Figure 4B). Therefore, both subunits of the heterodimeric Ku complex are required for viability when wild-type Pif1p is overexpressed. While this paper was being prepared for publication, another group reported that Pif1p overexpression causes poor growth in *yku70*Δ and *yku80*Δ cells [32].

### Excess Pif1p Is Lethal only in Strains with Long G-Tails

In addition to its role at telomeres, Ku is also required for nonhomologous end-joining (NHEJ) (reviewed in [33]). To determine if the lethal effects of Pif1p overexpression in *yku*Δ cells are related to the role of Ku in NHEJ, we overexpressed Pif1p in a *lig4*Δ strain (Figure 4C). *LIG4* encodes a ligase that is required for NHEJ but that does not affect telomeres [34,35]. As Pif1p overexpression did not reduce viability of *lig4*Δ cells (Figure 4C), its effects on viability of *yku*Δ cells must be independent of NHEJ.

Another possibility is that overexpression of Pif1p, which results in modest telomere shortening [21],would be lethal in any strain that has short telomeres. The ATM paralog Tel1p is a checkpoint kinase in S. cerevisiae that has a major role in telomere maintenance. Telomeres in *tel1*Δ cells are very short but stable [36] with normal length G-tails [37] and wild-type levels of Cdc13p binding [38]. However, Est2p and Est1p telomere binding is markedly reduced in *tel1*Δ cells, especially in late S/G2 phase [38]. If Pif1p overexpression results in lethality in any strain with short telomeres, then overexpression of Pif1p should cause lethality in *tel1*Δ cells. Contrary to this expectation, Pif1p overexpression did not reduce viability in *tel1*Δ cells, although it did reduce growth rate (as it did in wild-type cells) (Figure 4D).

If the lethality of *yku*Δ strains that overexpress Pif1p results from the synergistic effects of reduced telomere end protection and reduced levels of telomere-associated telomerase, then overexpression of Pif1p might not affect viability in a strain that already lacks G1 phase telomerase but has normal end protection. The *tlc1*Δ*48* and *yku80-135i* mutations eliminate interaction between Yku80p and *TLC1* telomerase RNA [16,17], and these strains have no Est2p at telomeres in G1 phase and reduced telomere-associated Est2p and Est1p in late S/G2 phase [16]. However, both strains have wild-type levels of Cdc13p and Ku telomere binding [16], and both have wild-type or near wild-type length G-tails [15,17,39]. Overexpression of Pif1p was not lethal in either *tlc1*Δ*48* (Figure 4A) or *yku80-135i* (Figure 4B) cells. In contrast, overexpression of Pif1p resulted in reduced viability of *yku80–1* and *yku80–4* cells (Fig 4E), two mutants that have longer G-tails than wild-type cells and partial defects in end protection [39].

### Overexpression of Pif1p Results in Reduced Viability of *cdc13–1* Strains at Permissive Temperatures

Of the strains examined, Pif1p overexpression reduced viability only in *yku* mutants that have longer G-tails. To determine if Pif1p overexpression is lethal in other strains with long G-tails, we examined the effects of Pif1p overexpression in *cdc13–1* cells. Cells carrying the temperature sensitive *cdc13–1* allele die at the restrictive temperature due to accumulation of single-stranded telomeric DNA but are viable at 23 °C [10]. A *cdc13–1* strain was transformed with an empty plasmid or the same plasmid containing *GAL*-*PIF1* or *GAL*-*pif1*-*K264A,* plated to glucose or to galactose plates and incubated at 23 °C to monitor growth. As seen previously, wild-type cells were viable but slow growing when Pif1p was overexpressed (Figure 5). However, overexpression of wild-type Pif1p (but not Pif1p-K264A) resulted in lethality of *cdc13–1* cells at the normally permissive temperature of 23 °C. As expected, all of the strains had wild-type growth rates when grown on glucose medium at 23 °C (Figure 5, right panel).

### Deletion of *EXO1* Restores Viability to *yku70*Δ Cells Overexpressing Pif1p and Partially Restores Viability to *cdc13–1* Cells Overexpressing Pif1p

A *yku70*Δ or *yku80*Δ strain is viable at 30 °C but is unable to form colonies at 37 °C [40,41]. This temperature sensitivity is likely due to telomere defects as it can be suppressed by overexpression of telomerase subunits [42], by amplification of subtelomeric repeats [43], or by deletion of *EXO1* [8,44]. Deletion of *EXO1* also suppresses the temperature sensitivity of *cdc13–1* cells [8,44]. We reasoned that removal of telomerase from telomeres in Pif1p overexpressing cells may allow nucleases such as Exo1p increased access to telomeric DNA. To test this model, we examined the effects of Pif1p overexpression in *yku70*Δ or *cdc13–1* cells that were also deleted for *EXO1*. If the model is correct, loss of Exo1p should relieve the lethality associated with excess Pif1p in these backgrounds.

**Figure 6:**
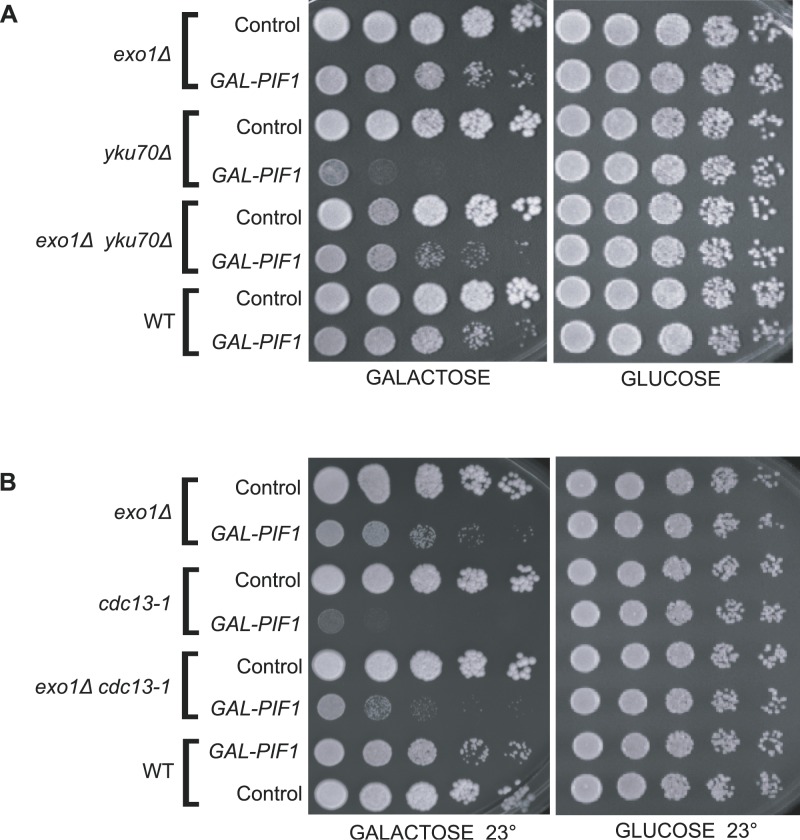
Deleting *EXO1* Reduces Lethality Caused by Pif1p Overexpression in *yku70*Δ and *cdc13–1* Cells Tetratype tetrads derived from diploids heterozygous for both *exo1*Δ and *yku70*Δ (A) or *exo1*Δ and *cdc13–1* (B) and containing the indicated plasmids (as described in Figure 3 legend) were grown overnight in selective liquid media containing glucose at 30 °C (A) or 23 °C (B). Cultures were serially diluted in water and spotted onto selective galactose (left) or glucose (right) plates and incubated at 30 °C for 5 d (galactose) or 2 d (glucose [A]) or at 23 °C for 7 d (galactose) or 5 d (glucose [B]).

Diploids heterozygous for *EXO1* and *YKU70* deletions were sporulated and dissected. Tetratype tetrad spores were transformed with an empty plasmid or with *GAL*-*PIF1.* We determined the ability of the various strains to form colonies on galactose and glucose plates at 30 °C. As predicted by the model, *exo1*Δ *yku70*Δ cells overexpressing Pif1p formed colonies on galactose plates (Figure 6A, left panel). Thus, the loss of viability seen in *yku70*Δ strains upon Pif1p overexpression requires the activity of Exo1p. Likewise, deletion of *EXO1* was able to partially restore growth to *cdc13–1* cells overexpressing Pif1p (Figure 6B, left panel). The fact that the rescue was not complete in the *cdc13–1* background may reflect the fact that an as yet undiscovered nuclease acts in concert with Exo1p to generate telomeric single-stranded DNA in *cdc13–1* cells [8,44]. The suppression of the inviability in Pif1p overexpressing *yku70*Δ and *cdc13–1* cells by *EXO1* deletion argues that the loss of viability is due to defects in telomere structure.

### Loss of Pif1p Results in Partial Suppression of the Temperature Sensitivity of *yku70*Δ and *cdc13–1* Strains

Overexpression of Pif1p results in shortened telomeres and less telomere-bound telomerase, while Pif1p depletion results in long telomeres and more telomere-bound Est1p [20–22]. Here we show that Pif1p overexpression caused lethality in strains that have long G-tails (Figures 3–5). If overexpression of Pif1p in *yku*Δ and *cdc13–1* cells leads to inviability by exacerbating the reduced end protection in these strains, then depleting cells of Pif1p might relieve the temperature sensitivity of *yku70*Δ and *cdc13–1* cells.

**Figure 7:**
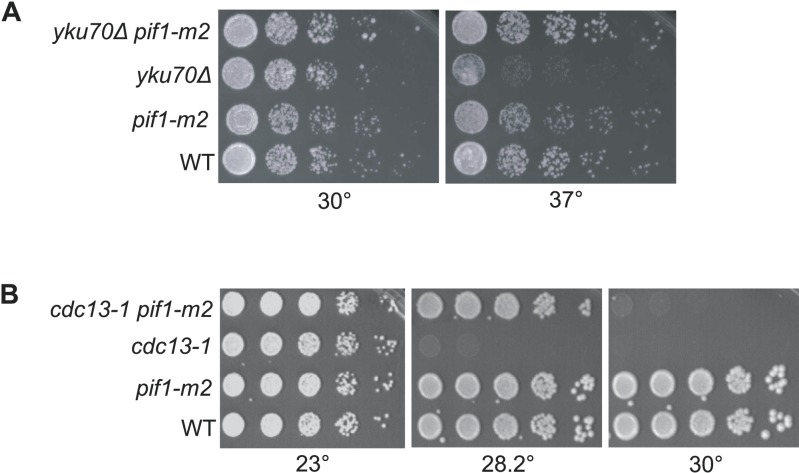
Deletion of Nuclear Pif1p Suppresses the Temperature Sensitive Growth of *yku70*Δ and *cdc13–1* Cells Tetratype tetrads were derived from diploids heterozygous for both *pif1-m2* (these cells express only the mitochondrial form of Pif1p) and *yku70*Δ (A) or both *pif1-m2* and *cdc13–1* (B). Cells were grown in complete liquid media overnight at 23 °C, then serially diluted in water, spotted onto YEPD plates, and incubated at the indicated temperatures for two days, except for the cells grown at 23 °C, which were incubated for three days.

To test this possibility, we introduced the *pif1-m2* allele into *yku70*Δ and *cdc13–1* cells. A *pif1-m2* strain expresses mitochondrial but not nuclear Pif1p [20,21]. We used *pif1-m2* since *pif1*Δ strains exhibit a petite phenotype that would impair our ability to monitor cell growth [20]. Heterozygous diploids were sporulated and dissected. Tetratype tetrads were identified, spotted onto rich medium, and incubated at various temperatures to assay growth. As expected, *yku70*Δ cells showed robust growth at 30 °C (Figure 7A, left) and poor growth at 37 °C (Figure 7A, right). The wild-type and *pif1-m2* control strains grew well at both temperatures. The double mutant *yku70*Δ *pif1-m2* cells also grew well at both temperatures (Figure 7A). Likewise, *cdc13–1 pif1-m2* cells grew well at 28.2 °C (Figure 7B, center), while *cdc13–1* cells did not grow at 28.2 °C (center panel). (As this paper was being prepared for publication, another group identified *pif1*Δ in a genome-wide screen for mutations that partially suppress the temperature sensitivity lethality of *cdc13–1* cells [45]). This suppression of temperature sensitivity was not complete as *cdc13–1 pif1-m2* cells did not grow at 30 °C (Figure 7B, right). Thus, depleting *yku70*Δ or *cdc13–1* cells of nuclear Pif1p improved their ability to grow at high temperatures.

### Disruption of the Ku-TLC1 RNA Interaction Exacerbates the Temperature Sensitivity of *cdc13–1* Strains

**Figure 8:**
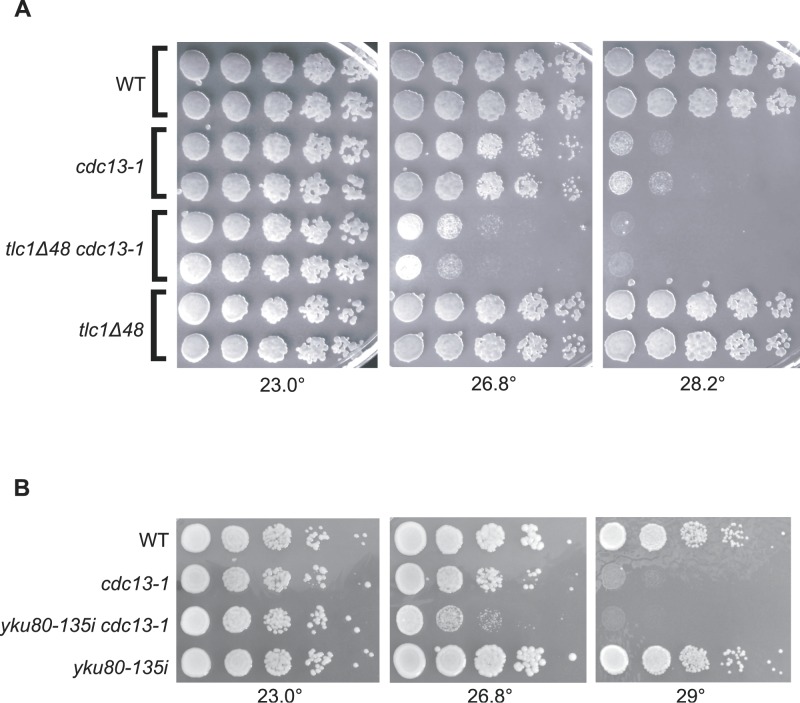
Disruption of the Ku-Telomerase RNA Interaction Exacerbates the Temperature Sensitive Growth of *cdc13–1* Strains Spore clones of the indicated genotypes from tetrads derived from diploids heterozygous for both *cdc13–1* and *tlc1*Δ*48* (A) or *cdc13–1 and yku80-135i* (B) were grown in complete liquid media for 2 d at 20 °C then serially diluted in water, spotted onto YEPD plates, and incubated at the indicated temperatures for 2 d (26.8 °C and 28.2 °C) or 3 d (23 °C and 26.8 °C).

To test our model that reduced levels of telomere-bound telomerase compromise viability in strains defective for telomere end protection, we examined the effects of eliminating the Ku/TLC1 RNA interaction using either the *tlc1*Δ*48* or *yku80-135i* allele in a *cdc13–1* strain. In both *tlc1*Δ*48* and *yku80-135i* cells, the telomere association [16] and end-protection activity [17] of Ku are not compromised, but the recruitment of Est2p to telomeres is lost in G1 phase and reduced in late S/G2 phase [16]. If the G1 pathway for recruiting Est2p to telomeres contributes to end protection, the *tlc1*Δ*48* and *yku80-135i* alleles should exacerbate the temperature sensitivity of *cdc13–1* cells. We constructed *cdc13–1 tlc1*Δ*48* and *cdc13–1 yku80*-*135i* double mutant strains and compared their growth at various temperatures to both wild-type and single mutant strains. Both double mutants were more temperature sensitive than the *cdc13–1* single mutant strain (Figure 8). Thus, reducing telomere association of telomerase using *tlc1*Δ*48, yku80-135i*, or Pif1p overexpression reduces the viability of *cdc13–1* cells.

## Discussion

We show that the nuclear form of the Pif1p helicase, which removes telomerase from DNA ends in vivo and in vitro [22], is a cell cycle-regulated protein. This regulation is achieved both by regulated accumulation of its mRNA [46] (a result also confirmed in our work, unpublished data) and by APC dependent regulation of Pif1p (Figures 1 and S1). As a result, the nuclear form of Pif1p peaked in abundance at about the same time in the cell cycle that telomerase lengthens telomeres (Figure 1) [12]. Restricting the expression of nuclear Pif1p to late in the cell cycle may facilitate the G1 phase telomere association of Est2p.

The cell cycle pattern of Pif1p abundance is consistent with telomere length analyses that indicate that endogenous Pif1p removes telomerase from late S/G2 phase telomeres (Figure 2). Deleting Pif1p resulted in longer telomeres in three strains, *yku80-135i, tlc1*Δ*48,* and *yku70*Δ, in which telomerase is telomere associated only in late S/G2 phase [16]. Thus, endogenous Pif1p must be able to act on the catalytically active form of telomerase that exists only transiently, late in the cell cycle. This conclusion is also supported by our previous finding that deleting *PIF1* results in more telomere-associated Est1p [22], which is telomere associated only in late S/G2 phase [14]. Endogenous Pif1p may also act on telomerase that is recruited to the telomere in G1 phase via a Ku-TLC1RNA interaction, since telomere elongation in the absence of Pif1p was not as great in *tlc1*Δ48 or *yku70*Δ cells as it was in wild-type cells (Figure 2). In addition, during the formation of new telomeres, Pif1p almost surely acts on Ku-recruited telomerase since the large increase in de novo telomere addition seen in *pif1*Δ cells is virtually eliminated in the absence of Ku [23].

The pattern of cell cycle-regulated abundance of nuclear Pif1p is similar to that observed for Est1p. In cells synchronized with alpha factor, Est1p is present in low amounts in late G1 phase and peaks in abundance in late S phase [14,16,47]. This pattern is achieved by both regulated mRNA accumulation [46,48] and proteasome dependent Est1p degradation [47]. The pattern and mechanism of cell cycle-regulated abundance of yeast Pif1p is also remarkably similar to that seen for the human PIF1-like protein, hPIF1 [49]. Like yeast Pif1p, hPIF1 abundance is controlled at both the mRNA and protein levels, and hPIF appears to be a direct target of the APC. In addition, hPIF is associated in vivo with TERT, the catalytic subunit of human telomerase. Likewise, in S. cerevisiae, mutations in the finger domain of Est2p reduce the sensitivity of telomere length to Pif1p, suggesting that the two yeast proteins also interact in vivo [50].

Overexpression of baker's yeast Pif1p from the non-cell cycle-regulated *GAL1* promoter results in reduced Est2p and Est1p at telomeres and elevated Cdc13p binding [22]. High Cdc13p binding is seen in strains with long G-tails [14,16] and low Cdc13p binding is seen in a strain with short G-tails [37,38]. Thus, elevated Cdc13p binding in Pif1p overexpressing cells suggests that Pif1p action exposes telomeres to degradation, which generates long G-tails. Excess Pif1p affected growth rate but had very little or no effect on viability of wild-type, *tel1*Δ, *tlc1*Δ*48,* or *yku80-135i* cells (Figure 4). All of these mutant strains have short telomeres but normal, or near normal, length G-tails [17,37,39]. In contrast, Pif1p overexpression reduced viability in five strains *(yku70*Δ, *yku80*Δ, *cdc13–1, yku80–1,* and *yku80–4)* (Figures 3–5), in which G-tails are longer and end protection is reduced compared to wild-type cells [6,10,39]. Since this loss of viability required the enzymatic activity of Pif1p (Figures 3–5, *pif1-K264A*), the deleterious effects of Pif1p overexpression are unlikely due to the titration of a structural protein(s) away from telomeres. We also reduced the levels of telomere-associated Est2p by a method that did not involve changing Pif1p levels. We introduced the *tcl1*Δ*48* or the *yku80-135i* allele into the *cdc13–1* strain (Figure 8). Both of these alleles eliminate the interaction between TLC1 RNA and Ku, an interaction that is required to bring Est2p to the telomere in G1 phase and for wild-type levels of telomerase in late S/G2 phase but does not affect Ku or Cdc13p telomere binding [16,17]. Both the *cdc13–1 tcl1*Δ*48* cells and the *cdc13–1 yku80-135i* cells were more temperature sensitive than *cdc13–1* cells (Figure 8). Thus, removing Est2p from telomeres in G1 phase and/or reducing telomerase levels in late S/G2 phase by mutation or by Pif1p overexpression is deleterious to telomere function but only in strains where reduced telomere end protection leads to long G-tails.

A possible explanation for the effects of Pif1p overexpression and of the *tlc1*Δ*48* and *yku80-135i* alleles on the viability of *cdc13–1* and *yku*Δ cells is that combining either of these mutations with any condition that causes telomere shortening exacerbates their inherent defects in telomere capping. However, deleting *TEL1* results in very short telomeres [36], yet *tel1*Δ *cdc13–1* and *tel1*Δ *yku*Δ cells are viable [9]. In addition, when *TEL1* is deleted in a *cdc13–1* or *yku*Δ strain, compared to the single mutant, the capping defect is either unchanged (*cdc13–1 tel1*Δ) or modestly increased (*yku*Δ *tel1*Δ). We conclude that reducing the levels of telomere-associated Est2p is lethal in strains that have a defect in telomere capping that is manifest by increased access to nucleases and hence longer G-tails. This interpretation is supported by the finding that deleting *EXO1*, a 5′ to 3′ exonuclease that acts at telomeres [8,11], suppressed the lethal effects of Pif1p overexpression in *yku70*Δ and *cdc13–1* cells (Figure 6). Even endogenous levels of Pif1p must further decrease the already compromised telomere function of *cdc13–1* and *yku70*Δ cells since depleting nuclear Pif1p in these backgrounds improved viability in both strains (Figure 7).

There are two general models that can explain why reduced Est2p telomere binding impairs viability and increased Est2p improves viability in strains with long G-tails. The first model suggests that when G-tails are long, reducing the amount of telomere-bound Est2p makes it harder to lengthen telomeres. For example, when G-tails are long and telomerase is limiting, it may be difficult to position Est2p at the very end of the G-tail, which is essential for it to extend the 3′ terminus. This model is supported by the finding that the number of telomerase complexes in yeast is low, perhaps as low or lower than the number of telomeres per cell [51]. Indeed, Est2p is haplo-insufficient in diploid *yku80*Δ cells (J.A. Phillips, L.R. Vega, and V.A. Zakian, unpublished data). The second model suggests that the pathway that recruits Est2p to telomeres in G1 phase and that is required for wild-type levels of telomere-associated telomerase in late S/G2 phase contributes to telomere end protection. In this model, the role of Est2p in end protection would be dispensable except in strains in which end protection is already reduced. A structural role for telomerase in end protection is consistent with results in other organisms. For example, in the yeast *Candida albicans,* deleting telomerase subunits results in very rapid telomere shortening, much faster than that expected from incomplete replication alone [52], and ectopic expression of enzymatically compromised TERT in cultured human cells can extend their replicative lifespan without increasing telomere length [53–55].

## Materials and Methods

### Strains and growth conditions.

All experiments were carried out in the YPH499 background (*MAT*a *ura3–52 lys2–801 ade2–101 trp1-*Δ*63 his1-*Δ*200 leu2-*Δ*1* [56]) except the *pif1-m1* and *pif1-m2* synchronies and the APC experiments, which were carried out in the W303 background (*MAT*a *leu2–3,112 trp1–1 can1–100 ura3–1 ade2–1 his3–11,15* [57]). For Pif1p overexpression experiments, strains were transformed with either the pSH380 plasmid (empty vector control, a pRS315-derived *CEN* plasmid [56,58]) or pVS45, which is pSH380 containing the nuclear form of Pif1p under the control of the *GAL1* promoter, or with pGPKA, which is the same as pVS45 except that the *PIF1* gene contains the K264A mutation in the Walker A box, which inactivates the ATPase/helicase activity of Pif1p [21]*.* The *pif1*Δ, *pif1-m1*, *pif1-m2* [20], and *lig4*Δ [59] strains were as described. The *exo1*::*URA3* deletion was created using pMPY 3xHA as described (see Table 1 for sequence of primers) [60]. After PCR amplification, the *EXO1* deletion fragment was gel purified and used to transform YPH499 and YPH500 (these two strains are isogenic except at the *MAT* locus). Transformants were selected on complete synthetic media lacking uracil, and integration was verified by Southern blot analysis. Deletion of *YKU80* was done in a similar manner (see Table 1 for primers). The *cdc13–1* mutation [10] was introduced by multiple backcrosses into the YPH499 background by A. Taggart. *yku70*Δ::*HIS3* [61] or *cdc13–1* cells were mated to *exo1*Δ::*URA3* cells. Cells were sporulated, tetrads were dissected, and tetratype tetrads were identified by the segregation of appropriate genetic markers. Each spore clone was transformed independently with pSH380, pVS45, and pGPKA, and assayed for growth on plates lacking leucine containing either 3% galactose or 2% glucose. Two individual tetratype tetrads were assayed for growth. Cells bearing the *pif1-m2* allele were crossed to either *yku70*Δ::*HIS3* or to *cdc13–1* strains at 23 °C, diploids were selected and restreaked two to five times prior to sporulation to equilibrate telomere length. *tlc1*Δ*48, yku80-135i* (generously provided by D. Gottschling)*, yku80–1,* and *yku80–4* (generously provided by A. Bertuch and V. Lundblad) alleles have been described [17,39,62]. Construction of *apc*Δ *(pds1*Δ, *clb5*Δ, *SIC1^10X^*, *apc2*Δ, *apc1*Δ, *cdc20*Δ, and *cdh1*Δ*)* and APC^+^ strains (*pds1*Δ, *clb5*Δ, and *SIC1^10X^ APC* ^+^ *)* has been previously described [28].

**Table 1:**
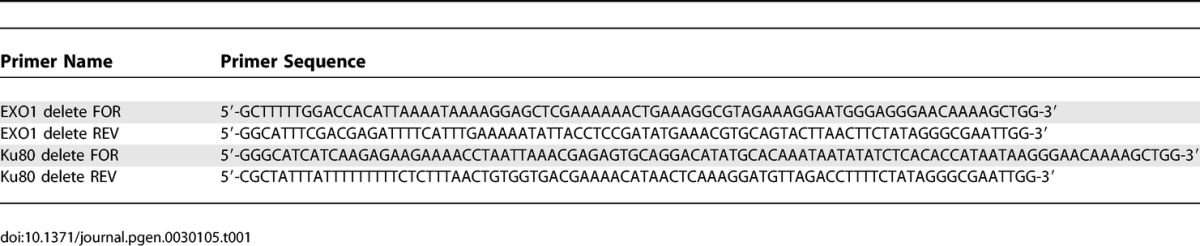
Primers Used for Deletion of *EXO1* and *YKU80*

### Synchrony experiment.

Wild-type, *pif1-m1,* and *pif1-m2* strains were grown overnight in YEPD media at 30 °C to log phase. The cultures were diluted to OD_660_ of 0.15 and arrested with 9 μg/ml alpha factor at 23 °C for 4 h. At T = 0, cultures were washed and resuspended in YEPD containing 20 μg/ml pronase. At the indicated time points, cells were harvested for protein, RNA, and FACS analysis by adding NaN_3_/EDTA. Equivalent cell numbers were harvested at each time point. For western blot analysis, protein was harvested by TCA treatment [63], and protein yields were quantitated by Bradford assays (Bio-Rad, http://www.bio-rad.com). We subjected 25 μg of total protein to SDS-PAGE analysis on 10% gels. After transfer onto nitrocellulose membranes, blots were blocked in 5% NFM/TBST 0.5% Tween 20 overnight and then probed for 3 h with antibodies raised against the amino terminus of Pif1p [64]. Protein extracts for APC experiments were prepared as described in [28]. For FACS analysis, cells were analyzed in a FACScan single laser fixed-alignment benchtop analyzer with an emission at 488 nm. A 530/30 band-pass filter was used to collect emissions in front of the detector.

### Pif1p half-life assay.

APC^+^ and *apc*Δ strains were grown to log phase at 23 °C and treated with 50 μg/ml cycloheximide. Equivalent OD of cells were collected for western blot, and samples fixed in 70% ethanol for flow cytometery at increasing time points. Protein extracts were made by first boiling cells in preheated SDS sample buffer (50 mM Tris [pH 7.5], 5 mM EDTA, 5% SDS, 10% glycerol, 0.5% β-mercaptoethanol, bromophenol blue, 1 μg/ml leupeptin, 1μg/ml bestatin, 1 mM benzamidine, 1 μg/ml pepstatin A, 17 μg/ml PMSF, 5 mM sodium fluoride, 80 mM β-glycerophosphate, and 1 mM sodium orthovanadate) for 5 min. Following boiling, glass beads were added and samples were subjected to bead beating for 3 min in a Mini BeadBeater (Biospec, http://www.biospec.com). Extracts were subjected to SDS-PAGE on 9% gels and analyzed by western blot with antibodies against Pif1p [64], Clb2p, and Cdc28p (Santa Cruz Biotechnology, http://www.scbt.com). For cell cycle analysis, fixed cells were sonicated, treated with 0.25 mg/ml RNaseA for 1 h at 50 °C, followed by digestion with 0.125 mg/ml Proteinase K for 1 h at 50 °C, and labeling with 1 μM Sytox Green (Invitrogen, http://www.invitrogen.com). Data were collected using a FACS Calibur (Becton Dickinson, http://www.bd.com) and analyzed with FlowJo (Tree Star, http://www.treestar.com) software.

### Cell viability assays.

To determine viability of liquid grown cells (Figure 3), wild-type or *yku70*Δ cells containing the indicated plasmids were grown overnight at 30 °C in defined medium lacking leucine (to select for the plasmid) containing 3% raffinose. At time zero, galactose was added to a final concentration of 3% w/v to induce protein expression. At the indicated time points, viability was monitored by plating a known number of cells (as determined by counting cells with a hemacytometer) onto selective plates containing glucose that were incubated at 30 °C. For the plating experiments in Figures 4–8, cells were grown in selective liquid media containing glucose overnight at 30 °C or at the indicated temperature. Cultures were serially diluted in water and spotted onto selective glucose plates and galactose plates and incubated at 30 °C or at the indicated temperature to monitor growth.

## Supporting Information

Figure S1 Pif1p Is Stabilized in *apc*Δ Cells APC^+^ and *apc*Δ cells were treated with cycloheximide (chx) for the indicated number of minutes (min chx), and levels of Pif1p and the established APC target Clb2p were analyzed by western blot. Short and long exposures of the blots are shown to allow comparison between the two cell types. Cdc28p is shown as a loading control. Cell cycle position was analyzed by flow cytometry at each time point, demonstrating that the cell cycle position was similar in APC^+^ and *apc*Δ strains.
(752 KB AI).

## Abbreviations

APC: anaphase-promoting complex
FACS: fluorescence-activated cell sorting
NHEJ: nonhomologous end-joining
