# Supplementary material for: Sensitivity of Yeast Strains with Long G-Tails to Levels of Telomere-Bound Telomerase
Source: PLoS Genet. 2007 Jun 22;3(6):e105. doi: 10.1371/journal.pgen.0030105 (PMC1892048; doi:10.1371/journal.pgen.0030105)
Supplement: Supplementary file 1 [file pgen.0030105.sg001.pdf]

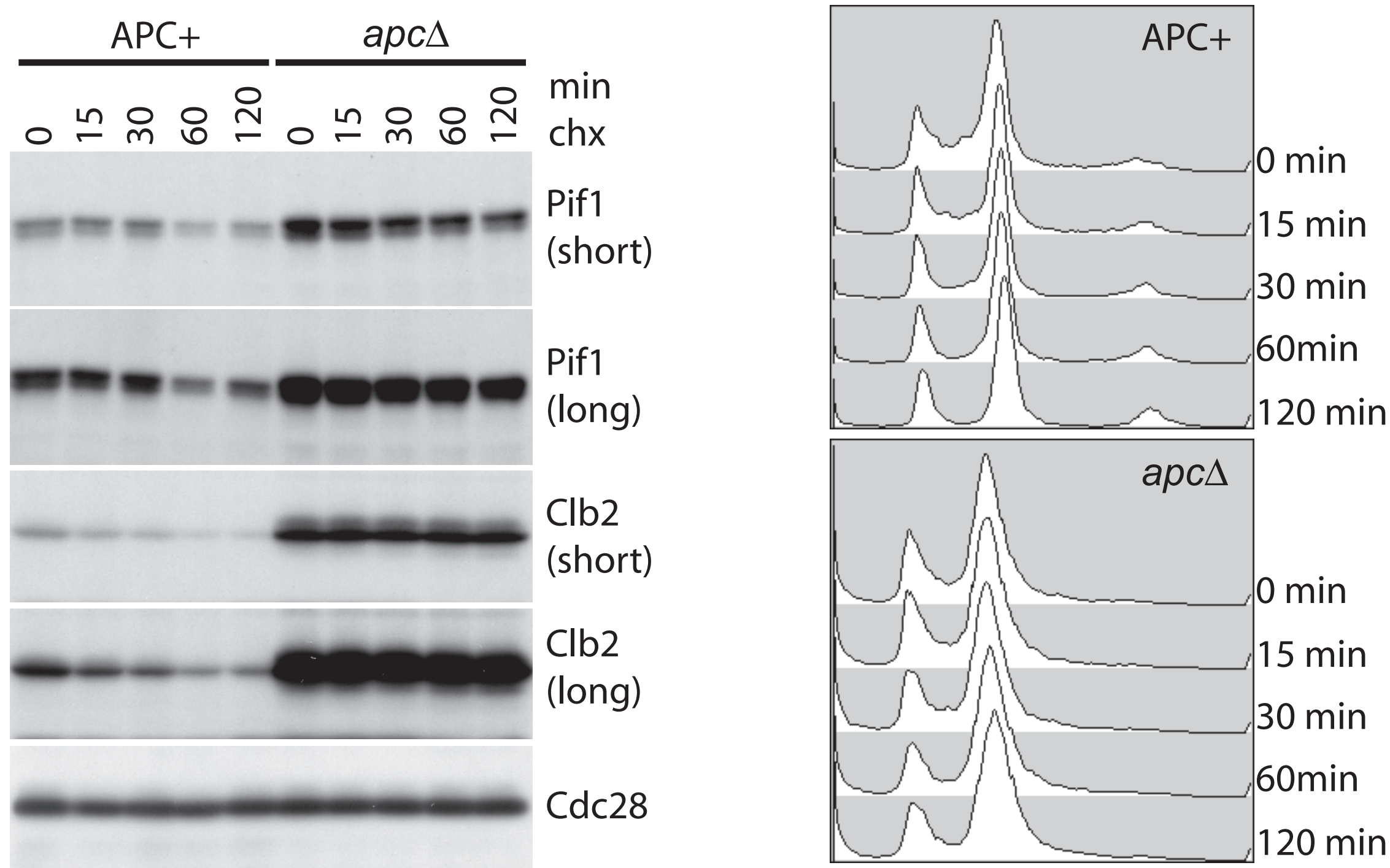

SUPPLEMENTARY FIGURE 1
Vega et al
